# Vestibulo-Ocular Reflex Stabilization after Vestibular Schwannoma Surgery: A Story Told by Saccades

**DOI:** 10.3389/fneur.2017.00015

**Published:** 2017-01-25

**Authors:** Angel Batuecas-Caletrio, Jorge Rey-Martinez, Gabriel Trinidad-Ruiz, Eusebi Matiño-Soler, Santiago Santa Cruz-Ruiz, Angel Muñoz-Herrera, Nicolas Perez-Fernandez

**Affiliations:** ^1^Otoneurology Unit, Department of Otorhinolaryngology, University Hospital of Salamanca, IBSAL, Salamanca, Spain; ^2^Skull Base Unit, Department of Otorhinolaryngology, University Hospital of Salamanca, IBSAL, Salamanca, Spain; ^3^Otolaryngology Unit ORL Gipuzkoa, Clinica Quiron, San Sebastian, Spain; ^4^Otoneurology Unit, Department of Otorhinolaryngology, University Hospital of Badajoz, Badajoz, Spain; ^5^Department of Otorhinolaryngology, Hospital General de Catalunya, Barcelona, Spain; ^6^Otoneurology Unit, Department of Otorhinolaryngology, Clínica Universidad de Navarra, University Hospital and Medical School, University of Navarra, Pamplona, Spain

**Keywords:** vestibular compensation, covert saccade, overt saccade, video head impulse test, PR score, vestibular schwannoma, caloric

## Abstract

**Objective:**

To evaluate vestibular compensation *via* measurement of the vestibulo-ocular reflex (VOR) following vestibular schwannoma surgery and its relationship with changes in saccades strategy after surgery.

**Patients:**

Thirty-six consecutive patients with vestibular schwannomas, without brainstem compression, underwent surgical resection. Patients were recruited from University Hospital of Salamanca, Spain.

**Methods:**

We assessed the age, sex, tumor size, degree of canalicular weakness, and preoperative video head impulse test (gain and saccade organization measured with PR score). Gain and saccade organization were compared with postoperative values at discharge and also at 1, 3, and 6 months. PR scores are a measure of the scatter of refixation saccades.

**Results:**

Patients with normal preoperative caloric function had higher PR scores (saccades were scattered) following surgery compared to patients with significant preoperative canal paresis (*p* < 0.05). VOR gain and the presence of covert/overt saccades preoperatively did not influence the PR score (*p* > 0.05), but a group of patients with very low VOR gain (<0.45) and covert/overt saccades before surgery had lower PR scores after surgery. The differences after 6 months were not significant.

**Conclusion:**

Patients with more severe vestibular dysfunction before vestibular schwannoma surgery show significantly faster vestibular compensation following surgery, manifested by changes in VOR gain and PR score. The scatter of compensatory saccades (as measured by the PR score) may be a surrogate early marker of clinical recovery, given its relationship to the Dizziness Handicap Inventory.

## Introduction

The head impulse test (HIT) was first described by Halmagyi and Curthoys in 1988 as a test of the vestibulo-ocular reflex (VOR) (1) and has become an established bedside assessment in the evaluation of the dizzy patient. It consists of providing a fast horizontal head thrusts while asking the subject to maintain gaze on a central target. Horizontal canal receptors ipsilateral to the direction of head movement are preferentially stimulated resulting in a reflexive eye movement with a low latency, similar velocity, and contrary direction to the head movement. In the presence of a peripheral vestibular deficit, the eyes move with the head and a “catch-up” saccade is required to bring gaze back toward the fixation target (2). The HIT is based on the detection of these “catch up” or refixation saccades.

The advent of the video HIT allowed online simultaneous recording of eye and head movements, facilitating measurement of the VOR gain (eye:head velocity) and identification of overt (occurring once the head movement is finished) (3) and covert (occurring while the head is still moving) (4, 5). In addition, the video HIT had allowed better qualitative and quantitative characterization of refixation saccades. The video HIT has a greater sensitivity and larger positive predictive value for vestibular dysfunction than the clinical HIT (6).

The taxonomy of refixation saccades captured during the video HIT is based on their latency relative to the onset of the head movement; each saccade can be analyzed individually and classified as either “overt” or “covert.” Another way to classify refixation saccades is based on the degree of synchrony of sequential refixation saccades. Using this method, saccades can be divided into two groups yielding either isochronic impulses (final look of the response is gathered) or asynchronic impulses (final look is scattered) (7). Interestingly, when the organization patterns of refixation saccades are considered, the scattered response correlates well with an uncompensated vestibular deficit in patients after surgery for a vestibular schwannoma and the gathered with a better clinical compensation (8).

Vestibular schwannoma is a slowly and irregularly growing benign tumor that induces a progressive vestibular function reduction. The main symptoms at diagnosis are hearing loss, imbalance, and tinnitus (9). The slow progressive reduction of vestibular function allows the gradual implementation of central adaptive mechanisms (vestibular compensation), which minimize vestibular schwannoma-related symptoms and influence vestibular examination (5, 10, 11–13).

After surgery for the definite and complete exeresis of the tumor, compensation has to proceed again and different mechanisms are involved, although the commissural network is one of the preferential sites to occur between the normal and damaged sides (14) and visual compensation will also contribute to vestibular compensation. In the initial postoperative phase, an intense vertigo as a manifestation of the acute modification in vestibular function is experienced in 23–49 to 66–78% of patients, leading to disequilibrium while central compensation begins to take place (15). This is paralleled by the reduction of spontaneous nystagmus intensity. Refixation saccades in the vHIT also cause temporal modifications and result in a shift from a combination of covert and overt saccades to mainly covert ones (16); this is mirrored in the observed change from scattered to gathered saccades.

Previous studies have shown the influence of vestibular damage before vestibular schwannoma surgery in vestibular compensation, showing that a severe vestibular deficit before surgery achieves a faster recovery of patients (17). This faster compensation has been measured with different objective tests such as subjective visual vertical or computerized dynamic posturography and semiquantitative measures such as the Dizziness Handicap Inventory (18).

We have previously studied long-term (>1 year) video HIT outcomes in a group of patients who underwent vestibular schwannoma surgery (8); the majority of patients had a “stabilized” video HIT morphology, with a gathered pattern of refixation saccades. A small group of patients had a scattered pattern in the refixation saccades. With the term “stabilization,” we mean the way (in the first and medium terms) to get the definite saccadic strategy to replace the absent VOR. A greater degree of scatter correlated with worse clinical outcomes (as measured with Dizziness Handicap Inventory).

The aim of the present study was to evaluate the short- and medium-term VOR characteristics and refixation saccade changes in patients undergoing vestibular schwannoma surgery.

## Patients and Methods

### Subjects

Thirty-six patients were diagnosed with a unilateral vestibular schwannoma who were prospectively scheduled for surgery between November 2011 and December 2015. All patients were subjected to retrolabyrinthine or translabyrinthine vestibular schwannoma surgery and were reviewed at 1, 3, and 6 months postoperatively.

Age, sex, tumor side, tumor size according to Koos’s classification (19), canal paresis, and video HIT findings were recorded. None of the patients underwent vestibular rehabilitation after surgery during the follow-up period. Written informed consent was obtained for all patients. Because of no new or exceptional interventions, local ethical committee approval was not needed.

The study was performed in accordance with the ethical guidelines of the 1975 Declaration of Helsinki. Patients with brainstem compression or other conditions that could affect postural control were excluded.

### Methods

#### VOR Assessment

The VOR was evaluated with the HIT. The physician stands behind the patient and grasps his or her head firmly with both hands. The patient is asked to keep looking at a stationary object on the wall that is at a distance of 90–100 cm. The head is quickly and unpredictably turned through 10–20° in the horizontal plane either to the left or the right, which permits testing of the corresponding horizontal semicircular canal. In order to register and measure head and eye velocity during the head impulse, we have used a video HIT system (vHIT, GN Otometrics, Denmark). The patient wears a pair of lightweight, tight-fitting goggles on which is mounted a small video camera and a half-silvered mirror that reflects the image of the patient’s right eye into the camera. The eye is illuminated by a low-level infrared light-emitting diode. A small sensor on the goggles measures the head movement. The whole goggle system weighs about 60 g and is secured tightly to the head to minimize goggle slippage. Calibration is performed and the procedure of vestibulo-ocular testing is initiated. The speed of head movement is measured by the sensor in the goggles, and the image of the eye is captured by the high-speed camera (250 Hz) and processed to yield eye velocity. At the end of each head turn, the head velocity stimulus and eye velocity response are displayed simultaneously on the screen so that the clinician can see how good the stimulus and response were, thereby providing a quick way to maximize the quality of the head impulse. Twenty impulses were delivered randomly in each direction. We evaluated the VOR mean gain (ratio of eye velocity to head velocity for every head rotation) and the appearance of saccades after head impulses to the right and left sides. The amplitude of the head rotation was 18–20°, and the peak head velocity of the impulse varied between 150 and 240°/s with a resultant acceleration of between 4500 and 7500°/s^2^.

#### HITCAL and PR Score

**Figure 1:**
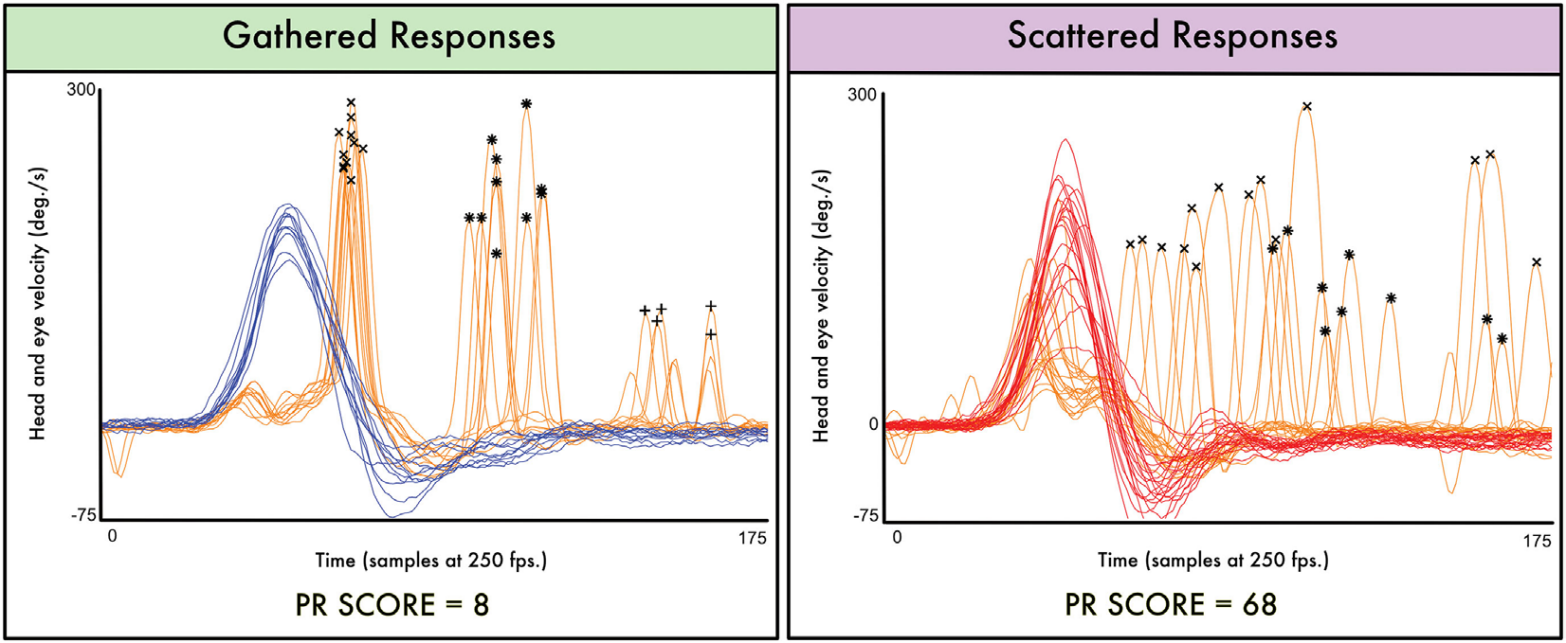
**Two real data cases are plotted to illustrate the PR score significance**. In the first (left) plot, the saccadic eye responses appear mainly on a narrow interval of time; in this gathered plot, the computed PR score has a low value (PR score = 8). In the second (right plot), the saccadic eye responses appear on a wide interval of time; even in some cases, the first responses appear after the second responses registered on other impulses; in this scattered plot, the computed PR score is much higher (PR score = 68). “X” mark is for the first group of detected saccades, “*” mark is for the second detected group, and “+” mark is for the third detected group. *Y* axis is head and eye velocity in degree per second, and *X* axis is the time domain measured in samples (at an approximated sampling frequency of 250 frames per second). Both plots and PR score measurements were obtained using the open source software HITCal 4.0 (www.mlibra.com).

Vestibulo-ocular reflex data were post processed using HITCal, a software tool designed to explore and analyze vHIT default output. Original results were exported from vHIT’s manufacturer software in eXtensible Markup Language format. We used HITCal to explore and obtain main variables from the records of each patient’s test: mean gain, mean head amplitude, mean peak of head velocity, and mean peak of head acceleration. We also used HITCal to obtain a PR score for each test—a parameter developed to measure the aggrupation of the saccadic responses appearance in the time domain. To obtain the PR score, HITCal uses a proprietary algorithm that is summarized in Table 1; first step in this algorithm is to detect the saccadic responses for each eye response; in the first step, the detected saccades are not classified using the covert/overt paradigm. In the PR score algorithm, saccadic eye responses are classified attending to the order of appearance after the positive peak of velocity in head impulse. With the detected saccadic responses, the algorithm computes the PR score for each group of appearance. The PR score is based on the coefficient of variation of the (time) moment of appearance of the peak eye velocity in the saccadic response computing all the eye responses present in all the impulses recorded in the same test. In the second step, the PR scores obtained for the first and second appearance groups are computed as main parameters of an arithmetic average. Finally, to get the global PR score, this value is multiplied by a scale factor to fit it in the numeric interval of 0–100. For each vHIT test, a low PR score result (near 0) means maximum gathered, high-grouped responses. A high PR score result (near 100) means maximum scattered, poor grouped responses. HITCal software development process and source code, and PR score algorithm and methodology have been previously published by our research group (7) (Figure 1; Table 1).

**Table 1:** **Automated saccade analysis and PR score algorithm**.

	Saccade recognition sequence	PR score algorithm
Step 1	Low pass filter of the head and eye graphs	*Pattern classification* logic condition If coefficient of variation of eye peak >12 for first saccades group or coefficient of variation >35 for second saccades group, result is scattered

Step 2	Relative max values recognition, using conditions	Group PR score
	Minimum velocity: 65°/s	Coefficient of variation of peaks in each group
	Minimum distance between peaks in eye graph: 15 samples	The group is determined by the order of appearance of saccades
	Minimum distance with head velocity peak: 10 samples	

Step 3	Outlier detection, only the two first peak values with +20 or −15 difference from mean of peaks was unmarked as saccades	*Global PR score* calculation
		2.5 × (0.8 × CV1 + 0.2 × CV2), where CV1 is coefficient of variation of first registered saccades and CV2 is coefficient of variation of second registered saccades
		Global PR score corrections
		If score is >100, the value is reset to 100
		If score is >35, the value is adjusted with the number of peaks detected: if only a few impulses have peaks over 35, these impulses have a low impact on the PR SCORE

*Main steps in saccade recognition sequence and the PR score algorithm. For time samples, a frame rate of 250 frames per second is assumed*.

### Groups

Patients were classified into three groups according to the preoperative degree of canal paresis: Group A are patients with a normal caloric test, Group B are patients with a partial canal paresis from 26 to 70%, and Group C are patients with severe canal paresis (more than 71%).

Patients were also classified according to video HIT gain parameters: Group A with normal gain (≥0.80) and Group B with abnormal gain (≤0.80). In order to distinguish between patients with a mild grade of vestibular damage and patients with a severe vestibular deficit, a secondary arbitrary division was performed among Group B patients, according to whether the gain was between 0.46 and 0.79 (“low gain”) or below 0.46 (“very low gain”).

Finally, patients were divided in terms of the preoperative presence of overt or covert saccades: “no saccades,” “only overt saccades,” “only covert saccades,” and “covert and overt saccades.”

### Statistical Analysis

All data were stored in an Excel file and analyzed using SPSS 21.0. Non-parametric tests were preferred to assess statistic significance of the differences found between quantitative variables due to sample size and Kolmogorov–Smirnov test’s results. Quantitative PR score values were compared before and after the operative procedures (Wilcoxon test) to assess correlation (ρ Spearman).

## Results

### Patients

From November 2011 to December 2015, 36 patients (21 females) were included in the study. Mean age for the total population was 52 ± 14 years. The affected side was the right in 20 patients and the left in 16 patients. There were no group differences for age, gender, or the affected side.

According to the tumor size, there were 4 grade I patients, 11 grade II patients, 17 grade III patients, and 4 grade IV patients. Surgical approaches were retrosigmoidal and translabyrinthine in 27 and 8 patients, respectively. Mean values of PR are shown in Table 2.

**Table 2:** **Mean PR values and SDs in the patient’s follow-up according to the different variables**.

Mean PR values			
**Caloric test**	**Normal**	**Moderate canal paresis**	**Severe canal paresis**

Pre	1.2 ± 0.6	5.6 ± 2.3	13.2 ± 4.5
Post 1	35.4 ± 7.2	30.5 ± 6.7	20.5 ± 5.6
Post 2	29.6 ± 6.1	22.9 ± 5.7	16.7 ± 5.1
Post 3	25.3 ± 4.9	18.4 ± 4.2	14.6 ± 4.3
Post 4	17.1 ± 4.1	15.6 ± 3.8	13.3 ± 3.6

**Gain**	**Normal**	**Low gain**	

Pre	0.6 ± 0.4	8.2 ± 3.4	
Post 1	36.6 ± 7.7	32.9 ± 8.7	
Post 2	28.7 ± 6.2	25.4 ± 7.0	
Post 3	24.2 ± 4.8	19.4 ± 6.1	
Post 4	18.3 ± 3.9	17.6 ± 5.6	

**Gain**	**Normal**	**Low gain**	**Very low gain**

Pre	0.6 ± 0.4	6.2 ± 2.8	16.4 ± 4.1
Post 1	36.6 ± 7.7	34.1 ± 9.1	25.3 ± 5.4
Post 2	28.7 ± 6.2	27.2 ± 7.8	20.1 ± 4.2
Post 3	24.2 ± 4.8	21.5 ± 5.6	18.4 ± 4.0
Post 4	18.3 ± 3.9	17.6 ± 5.1	14.3 ± 4.1

**Covert saccades**	**Yes**	**No**	

Pre	5.4 ± 2.3	0	
Post 1	36.2 ± 9.1	37.5 ± 8.3	
Post 2	26.6 ± 7.6	27.6 ± 7.3	
Post 3	21.8 ± 5.8	22.7 ± 5.1	
Post 4	15.7 ± 4.1	16.5 ± 4.2	

**Overt saccades**	**Yes**	**No**	

Pre	7.8 ± 3.4	0	
Post 1	32.6 ± 8.1	37.5 ± 8.3	
Post 2	24.5 ± 6.9	27.6 ± 7.3	
Post 3	19.7 ± 5.3	22.7 ± 5.1	
Post 4	15.3 ± 3.7	16.5 ± 4.2	

**Covert and overt saccades**	**Yes**	**No**	

Pre	16.8 ± 5.2	0	
Post 1	27.1 ± 7.7	37.5 ± 8.3	
Post 2	20.9 ± 5.5	27.6 ± 7.3	
Post 3	18.3 ± 3.8	22.7 ± 5.1	
Post 4	14.3 ± 3.2	16.5 ± 4.2	

### Caloric Test and PR Score

**Figure 2:**
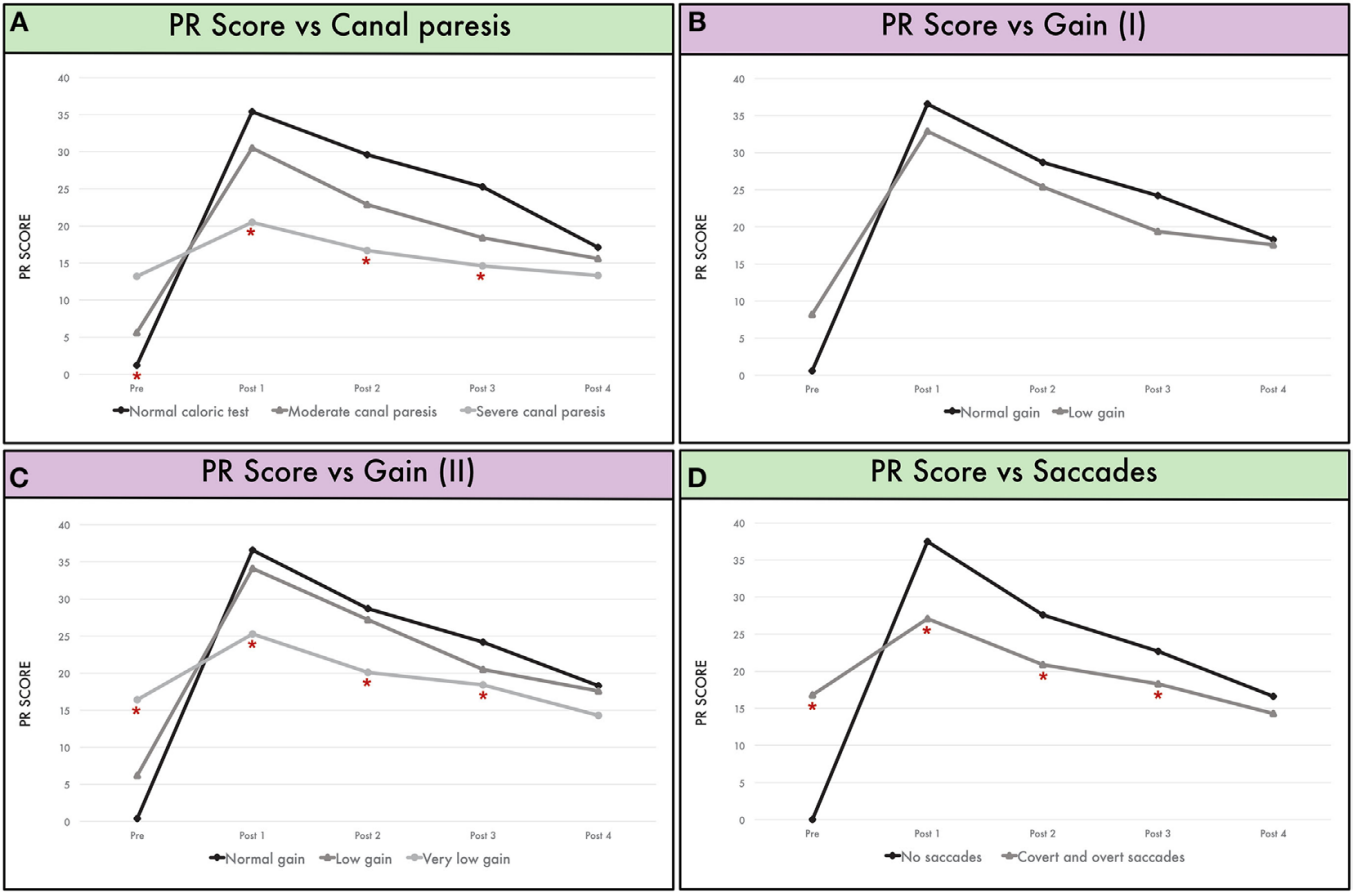
**(A)** Mean PR score according to canal paresis before surgery and during follow-up. Group A are patients with a normal caloric test, Group B are patients with a partial canal paresis from 26 to 70%, and Group C are patients with a severe canal paresis (more than 71%). **(B)** Mean PR score according to gain in the vHIT before surgery. Group A showing patients with normal gain (≥0.80) and Group B for low gain (<0.80). **(C)** Mean PR score according to gain in the vHIT before surgery. Group A showing patients with normal gain (≥0.80), Group B for low gain (<0.80 and >0.46), and Group C when gain <0.46 (“very low gain”). **(D)** Mean PR score in patients without saccades (Group “no saccades”) and patients with both covert and overt saccades (Group “covert and overt saccades”) before surgery.

We studied the influence of the preoperative caloric test function upon postoperative video HIT results (Figure 2A). Mean canal paresis for all patients involved in the study was 46 ± 42% (in the lesion side in all the patients). According to the criteria elicited in the material and methods section, 9 patients were included in Group A (0–20% canal paresis), 18 patients were included in Group B (21–70% canal paresis), and 9 patients were included in Group C (71–100% canal paresis).

Mean PR score for the population before surgery was 6.8. We found that greater preoperative canal paresis was associated with higher PR scores. Thus, the group with a normal caloric test before surgery (Group A) showed a PR score of 1.2. The group with a mild vestibular deficit (Group B) presented a mean PR score of 5.6, and the PR score before surgery in the group with a severe canal paresis was 13.2; differences between groups were statistically significant (*p* = 0.002).

The inverse relationship was observed postoperatively. Thus, immediately post-surgery, PR values were higher in the group with a normal caloric test before surgery (30.5; Group A) and compared to a PR value of 35.4 for the group with a mild canal paresis before surgery (Group B). Equally, PR score immediately after surgery in the group with a severe canal paresis before surgery (Group C) was 20.5, lower than Group B and Group A. Differences were significant between three groups in the first postoperative assessment (*p* = 0.005). In the follow-up (1 month after surgery, 3 months after surgery, and 6 months after surgery), PR values were reduced in the three groups but retained their group relationship such that they were always higher in Group A than Group B and Group C. However, differences were significant only at Post 1 (*p* = 0.005), Post 2 (*p* = 0.017), and Post 3 (*p* = 0.038) follow-up but not after Post 4, that is, 6 months after surgery, in which PR values were similar for the three groups.

### VOR Gain and PR Score

Mean VOR gain before surgery was 0.71 ± 0.26 for all patients in the affected side and 0.90 (0.90 ± 0.12) in the non-affected side.

Gain before surgery was considered normal (≥0.80) in 22 patients (Group normal) and abnormal (<0.80) in 14 patients (Group abnormal). Mean PR score in the group with normal gain before surgery was 0.6 (±0.3), and mean PR score in the group with abnormal gain before surgery was 8.2 (±1.4).

After surgery, mean gain for all patients was 0.31 (±0.08) in the operated side and 0.73 (±0.06) in the normal side.

No significant differences were observed in the PR score before and after surgery (at discharge, 1, 3, and 6 months) for either group (normal and abnormal gain before surgery) (*p* > 0.05) (Figure 2B).

Given such a large range of “abnormal VOR gain” (0.79–0.21), we created an arbitrary threshold (0.46) to separate patients with abnormal gain and a severe deficit (very low gain) from those with a mild deficit (low gain) (Figure 2C). Thus, PR score was higher before surgery in the group with “very low gain” (*p* = 0.009) and lower after surgery (*p* = 0.002 in Post 1, *p* = 0.017 in Post 2, and *p* = 0.035 in Post 3) than the groups with low gain or normal gain.

### Saccades and PR Score

The PR score was compared between patients with “no saccades” before surgery and those with “covert and overt” saccades before surgery (Figure 2D). Significant differences were observed in the pre-surgical evaluation (*p* = 0.012); these results were expected as from the definition of PR calculation. During follow-up, patients with refixation saccades before surgery had a significantly lower PR score at 1 week (*p* = 0.001), 1 month (*p* = 0.012), and 3 months (*p* = 0.02).

However, if the PR score is compared between patients showing isolated covert saccades before surgery (11 patients) with patients showing any saccade before surgery, no differences were obtained. The same situation was observed for patients showing isolated overt saccades (eight patients) (in both cases *p* > 0.05).

## Discussion

In our work, we were interested in the modification of the PR score after a definite vestibular loss in which a restoration of the VOR gain was not expected. This PR score reflects the amount of organization of refixation saccades across sequential head impulses and, indirectly, the level of vestibular disability of the patients as measured by the Dizziness Handicap Inventory (8). Saccadic reorganization is thus a useful marker of vestibular compensation following a complete unilateral vestibular ablation (18, 20). Although all patients suffer from dizziness after a vestibular schwannoma surgery, the manifestation of individual symptoms and therefore compensatory mechanisms is variable, despite a homogenous clinical syndrome (vestibular nerve transection). Our aim was to better understand these individual differences.

Our key finding is that patients with more severe vestibular dysfunction before schwannoma surgery show significantly faster vestibular compensation following surgery, manifested by changes in VOR gain and PR score. From a clinical perspective, a lower VOR gain and low PR score are more likely to cause oscillopsia during head movements, impacting negatively on the ability to carry out daily activities (21). One hypothesis is that patients with more severe preoperative canal paresis are more likely to induce central compensation (22) and thus recover more quickly post operatively (higher PR score). Second, we found a floor effect for PR scores over time; thus, regardless of preoperative canal dysfunction, there was a minimum PR score with associated refixation saccades that did not fully normalize. From a clinical perspective, such a PR score could be regarded as a target “recovery” value; it will be interesting to know in future works if this is the baseline for these patients, which represents eventually a good clinically compensated status. An elevated PR score (meaning heterochrony in refixation saccades between impulses) in the late postoperative phase would be an indicative of impaired vestibular adaptation, which is the necessary first step to compensation.

Vestibular compensation is an active process that in part impacts functionally upon the effectiveness of the VOR to stabilize gaze. When there is a chance of vestibular restoration after an acute vestibular failure (vestibular neuritis), both gain changes and saccade changes could influence the VOR contribution to vestibular compensation. But when vestibular failure is complete, this contribution can only be held by the changes in the saccades’ ability to compensate for the deficit of the VOR. The recovery of balance after vestibular schwannoma surgery is a slow process that begins early in the immediate postoperative phase and continues progressively but appears to be maximal during the first 3 months (23, 24).

When a vestibular loss is complete, visual cues both from peripheral and central vision are critical to improve the VOR (25). Indeed, in order to improve visual stabilization in relation to head movement, the combination of modifications in the amplitude and direction of compensatory saccades and their central pre-programing are fundamental components of vestibular compensation (26, 27). Therefore, the gain of the VOR reflects the raw vestibular signal, but refixation saccades represent the resource used to solve the problem when the gain is low. The origin of such saccades has not been fully elucidated, but it is believed that quick compensatory saccades are related to pontine reticular formation and controlled by the cerebellum, with involvement of the interpositus nucleus and the nodulus (28), although also presumably under higher order cortical influence.

Previous studies showed a strong correlation between preoperative caloric weakness and both Subjective Visual Vertical and Dizziness Handicap Inventory outcomes after surgery (18). Our finding further suggests that vestibular compensation occurs faster in patients with greater vestibular deficit before surgery (29).

The occurrence of the covert saccades was related to severity of vestibular hypofunction (25), and they are present during both predictable and unpredictable head rotations (30). Although their latency suggests that they are triggered by non-visual afferences and/or central pre-programing, they can reduce gaze error sizably for ipsilesional head rotations (25, 31) and definitely have a symbiotic relationship with the VOR, there being even an inverse relationship between their recruitment and amplitude and the VOR gain for passive head rotations (32).

Thus, the presence of covert saccades suggests that the vestibular dysfunction has been compensated and eye–head coordination anticipates movements in the real-life situation (16).

As expected, patients with both covert and overt saccades before surgery manifest low or very low VOR gain. Given that VOR adaptation in patients with low VOR gain and saccades before surgery is a slow process, this could explain the minimal changes observed in VOR and PR scores after surgery.

In our opinion, it is very important to understand how the patient compensates for the loss of the VOR, given its implications for vestibular rehabilitation and prevention of long-term disability. In light of our findings, one can predict that patients who compensate with a “gathered pattern” saccadic strategy, with covert saccades always with the same latency, will have lower levels of disability (8) and postural instability, compared to those with a “scattered” saccadic strategy.

This is a very important consideration because VOR adaptation and the organization of the refixation saccades into a gathered pattern could be artificially induced in patients who have not developed it naturally, and improves imbalance symptoms in patients with unilateral vestibular loss and vestibular disability. Indeed, it has been demonstrated that the close relationship between a lower value of PR and a better score in DHI is an indicative of better clinical outcomes (27).

In summary, we have shown that patients with more severe vestibular dysfunction before vestibular schwannoma surgery show significantly faster vestibular compensation following surgery, manifested by changes in VOR gain and PR score. Second, a thorough preoperative vestibular assessment in patients undergoing vestibular schwannoma surgery may identify early poor prognostic factors that could be addressed in the early postoperative stages to minimize clinical disability through promoting vestibular compensation.

## Author Contributions

AB-C, AM-H, and SR performed surgery and visited patients after surgery. AB-C tested all the patients with vHIT. AB-C, JR-M, NP-F, and GR designed the study, wrote much of the paper, and conducted the analysis. JR-M, NP-F, ES, and AB-C have developed HitCal and PR score.

## Conflict of Interest Statement

The authors declare that the research was conducted in the absence of any commercial or financial relationships that could be construed as a potential conflict of interest.
